# Energy Expenditure Combining Strength and Aerobic Training

**DOI:** 10.2478/v10078-011-0054-5

**Published:** 2011-10-04

**Authors:** José Vilaça, Martim Bottaro, Catarina Santos

**Affiliations:** 1 Department of Sport Sciences, Exercise and Health, University of Trás-os-Montes and Alto Douro (UTAD), Vila Real, Portugal; 2 Research Centre for Sport Sciences, Health and Human Development (CIDESD), Vila Real, Portugal; 3 University of Brasilia, Brasilia, Brazil

**Keywords:** Concurrent training, energy expenditure, oxygen consumption, resistance exercise, aerobic exercise

## Abstract

The combination of Strength Training (ST) with Aerobic Training (AT) exercises in the same training session, which commonly appears in literature as the concurrent training, is widely used in fitness and physical condition programs, especially when the aim is to increase the energy expenditure during and after training session. The aim of this study was to identify, through literature, whether the combination of exercises of the ST with exercises of the AT allows changes in body composition and energy expenditure during and after the training session. Chronic studies have showed a positive effect on body composition (decreased in relative body fat) when the ST are combined with AT. Similarly, the acute effects of the order of combining these two types of exercise does not seem to affect energy expenditure, measured by oxygen consumption (VO_2_), during the training session and only change this expenditure in the first 15 minutes after the training session. In conclusion, we can say that the studies indicate that the combination of exercises of the ST with exercises of the AT has a positive effect on changes in body composition, and energy expenditure during and after training sessions.

## Introduction

The concurrent training is widely used in fitness and physical condition programs, especially when the aim is to increase the energy expenditure during and after training session. However, it is not clear how the fatigue produced by a mode of exercise can adversely affect the quality and quantity of the other exercise mode (Lemos et al., 2009). Although the literature is scarce, this study will seek to present the acute and chronic effects of a combination of strength (ST) and aerobic training (AT) exercises on the energy expenditure during and after training sessions. It was conducted a systematic review of the literature based on articles that were indexed to the following databases: Medline, Sport Discus, ISI and Scielo. After the review the following inclusion criteria was used: i) chronic studies in which ST exercises were combined with the AT exercises, in the same training program, and the objective was to analyze their effect on morphological changes; ii) studies in which ST exercises were combined with the AT exercises, in the same training session, and the objective was to analyze the acute effect of concurrent training on energy expenditure during and/or after the training session; iii) data from your laboratory.

### Chronic Effects of Strength Training and Aerobic Training Exercises, in the Same Training Program, on Body Composition Changes

Several studies show positive chronic effects, on the energy expenditure and body composition, with the combination of ST exercise and AT exercise in the same training program (Dolezal and Potteiger, 1998; Balabinis et al. 2003;; Davis et al., 2009; Sillanpää et al., 2009). Although, only a few combine in the same session ST with AT exercises (Dolezal and Potteiger, 1998; Davis et al, 2009).

Dolezal and Potteiger (1998) reported an increased in absolute resting metabolic rate (RMR) in subjects when performed a combination of the ST with the AT, in the same training session, and when performed the ST alone. It was also observed a decrease in RMR when the subjects performed only the AT. However, when the absolute value of RMR was divided by fat-free mass there was no significant increase in either group (ST, ST+AT and AT). It is observed, in this study, that there was also a significant increase in lean body mass in subjects who practiced only the ST, and the subjects who practiced the concurrent training. Thus, revealing the importance of increased lean body mass to have an absolute increase in RMR. This fact becomes relevant in terms of body composition changes, since the RMR represents about 50% of daily energy expenditure in active individuals and 70% in sedentary (Wahrlich and Anjos, 2001). On this study the ST was performed before the AT.

Recently, Sillanpää et al. (2009), observed a reduction in percent body fat and increased lean body mass, in subjects who performed the combined training in relation to the subjects who performed only the ST, or the AT, and the control group. However, the subjects that combined the ST with the AT performed each session (ST or AT) on separate days, rising to double the training frequency (2 to 4) when compared to those who did only the ST or AT. Balabinis et al. (2003) also noted a decrease in relative body fat, with higher percent in subjects who combined the ST with the AT, compared with those who only performed the ST and AT (15.5%, 14.9% and 4.9%, combined, ST and AT, respectively). Note that all groups performed their training for 7 weeks, with four weekly sessions, and that the group that combined the ST with the AT performed the AT exercises in the morning and seven hours later the ST exercises. As on the study by Sillanpää et al. (2009), in the study performed by Balabinis et al. (2003) there is also a higher training volume, in the group who performed the combined training, in relation to the groups who performed the AT and ST alone. This higher volume and frequency may have influenced the results of these studies.

Davis et al. (2009), conducted a study which revealed a decrease in relative body fat in overweight young women using the combination of AT with ST. In this study the sets of ST exercises were performed in pairs, one exercise for the upper and another for the lower body, for 1 minute, after each set an AT exercise was performed for 2 minutes. The total of each component of ST and AT was 30 minutes. Therefore, the combination of AT exercise between blocks of ST seems to have a positive effect in reducing relative body fat.

We can thus conclude, based on the articles analyzed, that there is a chronic positive effect on the level of loss of relative body fat, with the combination of ST with the AT in the same training session (Dolezal and Potteiger, 1998; Davis et al., 2009) and in different sessions (Balabinis et al. 2003; Sillanpää et al., 2009), however when used in different sessions the training volume and frequency (number of sessions per week) may be a factor that may have influenced the results. The sequence of the combine the ST and AT exercises does not seem to change this positive effect.

### Acute effect of Concurrent Training on energy expenditure

The acute effects of combining the two types of exercises previously mentioned in the same training session on oxygen consumption (VO_2_) during and/or after the training session, are scarcely reported in the literature (Drummond et al., 2005; Lira et al., 2007; Monteiro et al., 2008; Kang et al., 2009).

Drummond et al. (2005) observed the sequence of AT and ST, performed on the same training session, on oxygen consumption after training session (VO_2_rec). Nevertheless, in the study mentioned above, the ST, when executed alone, caused a higher VO_2_rec than the AT/ST, ST/AT and AT groups. In your laboratory we also observed the effect of the sequence of the combination of an AT with ST exercises, in the same session, on the VO_2_ during training session and 30 minutes after the session. The exercise of AT was placed before ST exercises (BST) in the middle of two blocks of three ST exercises (MST) and after ST exercises (AST). We observed only significant differences in the values of VO_2_rec in periods of 5 to 15 minutes, between the BST and AST sequence and in the period 10 to 15 minutes between the MST and AST sequences. The AST sequence always presented lower VO_2_rec values than the others sequences. It is noted that the values of VO_2_rec in the data from your laboratory and in Drummond et al. (2005) remained above resting values 30 minutes after the end of the training session. But, as in our unpublished data, in the study of Drummond et al. (2005) only in the 15 minutes after training session was observed significantly differences between ST and AT sequences. Based on these data the combination of AT exercises with ST exercises only influences the response of VO_2_rec on the period of 5 to 15 minutes. Also, the sequence ST/AT presented the lowest VO_2_rec values.

In relation to VO_2_ during the training session, in the study performed by Drummond et al, (2005), it was observed that the AT exercise (running on a treadmill) when performed after ST shows a higher VO_2_ than when was performed before the ST or running alone (36,9±1,3; 37,6±1,3;38±1,3 e 39,3±1,3; 39,7±1,3; 40±1,3 ml.kg^−1^.min^−1^, 15, 20 e 25 minutes of exercise, before and after ST, respectively). Also, in a study performed by Kang et al. (2009), where were observed a significantly higher absolute VO_2_, in female subjects, between cycling in a cycle ergometer after ST exercise (3 sets of 8 reps of 6 exercises performed at 90% of the 8 RM and 3 sets of 12 reps of the 6 exercises performed at 60% of the 8 RM). Contrarily to Drummond et al. (2005) and Kang et al. (2009), on your unpublished data we not found significant differences during the AT exercise (cycling in a cycle ergometer), when this was followed or preceded of the ST exercises. A possible explanation of the differences between your data and Drummond et al. (2005) resides on the device used for the AT exercise (cycle ergometer vs. treadmill, respectively). It was been shown by Sedlock (1992) that the VO_2_ obtained in running on the treadmill is higher than cycling in the cycle ergometer, when was used the same exercise duration and intensity. Other factor that may have influenced the differences between the results of your data and Drummond et al. (2005), was that exercise on cycle ergometer was carried out with a full-seated position, thus mitigating the effect of gravity on the total body mass. Contrarily, when running on the treadmill the subjects had to bear the effect of gravity on their body mass and VO_2_ tended to be higher (McMurray et al., 2003). The sequence of ST exercises that were performed may have also affected the VO_2_ during AT. In your unpublished data the lower body muscle groups were interspersed with upper body exercise (upper limbs or middle zone of the trunk - the abdominal and lumbar). This exercise sequence was separated of no less than 11 minutes between lower limbs ST and subsequent AT exercise. This time period may have been large enough to allow for a recovery of lower limb muscle groups, possibly allowing a reduction of the phenomena of muscular fatigue of these muscles. This phenomenon could also help to explain the lower values of VO_2_ during the AT exercise in your unpublished data, when compared with those observed by Drummond et al. (2005). Indeed, in the study of Drummond et al., (2005), the order of exercises is based on the alternating ST exercises for the upper and lower body, excluding the exercises for muscle groups of the middle zone and was used an exercise involving the quadriceps femoris, who was performed immediately before the start of the running on the treadmill. As this muscle group interferes with the running, the response during the course of the treadmill running may have been influenced by this request, thus presenting a higher VO_2_ during its execution. It has been shown that the VO_2_ kinetics is faster and the O_2_ deficit is smaller for a given exercise when a previous bout of exercise is performed (Gerbino et al., 1996; Koppo and Bouckaert, 2000; Burnley et al., 2001; Scheuermann et al., 2001) involving the same muscle groups (Yoshida et al., 1995; Fukuba et al., 2002). The differences between Kang et al., (2009) and your unpublished data on the VO_2_ during AT exercise, may have been with the variations on the intensities during the AT exercise , in your data, and in Kang et al, (2009), the same exercise was performed with same intensity in all period.

Based on these data, we can say that the combination of ST exercises with the AT exercise may be affected by the performed order of ST exercises and the muscle group involved on these exercises. Also, the type of AT exercise and its manner of execution (Continuous vs. Intermittent) may also influence the VO_2_ during their execution. In relation to the energy expenditure during the ST exercises, when ST and AT training were combined in the same session, only in your unpublished data studied the VO_2_ component in the ST exercises and observed no significant differences in VO_2_ between the three orders used (BST, MST and AST). The mean values observed were between 17.73 and 16.43 ml.kg^−1^.min^−1^ in the ST exercises. These values were according the values of VO_2_ presented on the literature in this type of exercises in studies that present protocols of ST with the intensity and volume identical to the your unpublished, although using the ST as an only method of training (Hickson et al., 1984; Melanson et al., 2002).

Monteiro et al. (2008), tried to evaluate the introduction of running on treadmill, at 60% of maximum heart rate (HR) between each set of ST exercises (one set of 30s of ST exercise following by 30s of running on the treadmill and 10 to 15 seconds of rest). These authors compared the results with those obtained during the only execution of 60s of ST exercises, per set, with the same 10 to 15 seconds of rest between sets. The conclusions of the work were that the introduction of a treadmill run between the ST exercises caused a higher VO_2_ in the session than when was only performed the ST exercises (17.5 vs 20.8 ml.kg^−1^.min^−1^, in the female and 20.4 vs 23.8 ml.kg^−1^.min^−1^, in the male). These values of VO_2_ were according with the values of the study of your unpublished data (22.45, 22.34 and 21.4 ml.kg^−1^.min^−1^, BTF, MTF and ATF, respectively), which leads us to affirm that the combination of ST with AT exercises, seems to be a good method of increasing energy expenditure during the training session.

We can conclude, based on the studies analyzed, that the Aerobic exercises combined with Strength Training exercises did not interfere in the average VO_2_ during exercises execution and presented higher values of VO_2_ during and after exercise session. There is a chronic positive effect on the level of loss of relative body fat, with the combination of ST with the AT. We can also affirm that the sequence of the aerobic and the strength training exercises does not interfere in energy expenditure during the training session. Similarly, we can conclude that the inclusion of aerobic exercise after the Strength Training exercises reduces the VO_2_ measured during the first 15 minutes of recovery.

These findings allow the coaches a diversification on the training session planning that meets the preferences of the practitioners, lessen the training monotony and also allows a change in workout routine, without changing the main objective, increased energy expenditure.
